# Oncolytic vaccinia virus combined with radiotherapy induces apoptotic cell death in sarcoma cells by down-regulating the inhibitors of apoptosis

**DOI:** 10.18632/oncotarget.12820

**Published:** 2016-10-22

**Authors:** Michelle J. Wilkinson, Henry G. Smith, Gráinne McEntee, Joan Kyula-Currie, Tim D. Pencavel, David C. Mansfield, Aadil A. Khan, Victoria Roulstone, Andrew J. Hayes, Kevin J. Harrington

**Affiliations:** ^1^ Targeted Therapy Team, Division of Cancer Biology, The Institute of Cancer Research, London, UK; ^2^ Sarcoma/Melanoma Unit, Department of Academic Surgery, Royal Marsden Hospital NHS Foundation Trust, London, UK; ^3^ Division of Radiotherapy and Imaging, The Institute of Cancer Research, London, UK

**Keywords:** oncolytic virotherapy, radiation, soft tissue sarcoma

## Abstract

Advanced extremity melanoma and sarcoma present a significant therapeutic challenge, requiring multimodality therapy to treat or even palliate disease. These aggressive tumours are relatively chemo-resistant, therefore new treatment approaches are urgently required. We have previously reported on the efficacy of oncolytic virotherapy (OV) delivered by isolated limb perfusion. In this report, we have improved therapeutic outcomes by combining OV with radiotherapy. *In vitro*, the combination of oncolytic vaccinia virus (GLV-1h68) and radiotherapy demonstrated synergistic cytotoxicity. This effect was not due to increased viral replication, but mediated through induction of intrinsic apoptosis. GLV-1h68 therapy downregulated the anti-apoptotic BCL-2 proteins (MCL-1 and BCL-XL) and the downstream inhibitors of apoptosis, resulting in cleavage of effector caspases 3 and 7. In an *in vivo* ILP model, the combination of OV and radiotherapy significantly delayed tumour growth and prolonged survival compared to single agent therapy. These data suggest that the virally-mediated down-regulation of anti-apoptotic proteins may increase the sensitivity of tumour cells to the cytotoxic effects of ionizing radiation. Oncolytic virotherapy represents an exciting candidate for clinical development when delivered by ILP. Its ability to overcome anti-apoptotic signals within tumour cells points the way to further development in combination with conventional anti-cancer therapies.

## INTRODUCTION

Surgical resection is the first-line treatment for soft tissue sarcomas (STS), as it has the greatest curative potential. Radiotherapy has a well-established role in securing local disease control and may be given either pre- or post-operatively [1, 2]. However, approximately half of all patients with intermediate or high-grade STS will develop metastatic disease, with a 5-year survival of only 50% [1]. With the exception of specific chemo-sensitive subtypes, systemic chemotherapy has limited efficacy either as an adjuvant treatment or in the presence of metastatic disease [3, 4]. Therefore, new treatments and strategies to overcome treatment resistance are urgently needed.

A highly attenuated oncolytic vaccinia virus (VV), designated as GL-ONC1 (laboratory name GLV-1h68), has been developed [5]. Three expression casettes (RUC-GFP, LacZ, gusA) were inserted into F14.5L, J2R and A56R loci of the viral genome, respectively, resulting in attenuated virulence, enhanced tumour-specific targeting, and the ability to monitor viral transgene expression. *In vitro,* VV has single agent efficacy against a large range of tumour cell lines [5–13] including sarcoma [5, 7, 13].

The efficacy of the systemic administration of oncolytic virotherapy may be diminished by sequestration in the reticulo-endothelial system, clearance by circulating antibodies or an inability to penetrate the tumour in sufficient titres [14]. Intra-tumoural administration of virus has been shown to be effective in melanoma [15], although this route may be less practical in the presence of bulky, deep tumour deposits. Isolated limb perfusion (ILP) is a specialised surgical technique typically reserved for locally advanced or recurrent extremity sarcomas [16]. Due to the exclusion of the perfusion circuit from the systemic circulation, it was hypothesised that ILP would be an ideal delivery mechanism for oncolytic virotherapy, targeting delivery of the virus to the tumour whilst affording protection from sequestration. Previous work using an *in vivo* model of ILP confirmed the efficacy of this approach, with higher intra-tumoural viral titres achieved when VV was delivered by ILP compared with intravenous administration [17]. Furthermore, the addition of VV to standard ILP resulted in delayed tumour growth and prolonged survival [17].

Ionizing radiation is an important treatment modality for many solid tumours and can be used as single-agent therapy, in combination with radiosensitising chemotherapy or as an adjuvant treatment following surgical resection. Preclinical data indicate that the combination of oncolytic virotherapy (OV) and radiation therapy is promising, showing additional or synergistic anti-tumour effects *in vitro* and *in vivo* [6, 18–22]. These studies have resulted in translational phase I/II clinical trials [23, 24]. The mechanism of the interaction has not been fully elucidated but has the potential to be multifaceted: tumour-tropic viruses may act as radiosensitising agents, but radiation may also enhance viral immune stimulation and oncolysis by increasing viral uptake, replication, gene expression and cell death in irradiated cells [25]. However, the reported complex effects of radiation on viral infectivity, replication, gene expression and cytotoxicity mean that detailed mechanistic preclinical studies are an essential prerequisite to trials of new oncolytic viral agents in combination with radiation [6].

As previously stated, radiotherapy plays a vital role in the management of advanced extremity sarcoma. The possibility that OV may be able to radiosensitise these tumours is an exciting prospect but requires thorough initial *in vitro* and *in vivo* testing, due to the potential toxicities of combining these treatments. Assessing the compatibility of this novel treatment with radiotherapy may reveal exploitable synergistic relationships and discovery of a synergistic mechanism that may help in the development of combination treatment strategies that could potentially be used in the clinical setting [19]. The combination of VV and radiation has previously been shown to have beneficial therapeutic effects in preclinical studies in melanoma, head and neck cancers and glioma [6, 18, 19, 22]. GL-ONC1 is currently in clinical trial in combination with radiotherapy and cisplatin for locally advanced head and neck carcinoma (NCT01584284).

Whilst GLV-1h68 has been shown to have a potent anti-tumour effect, the precise mechanism of its cytotoxicity is still unclear. Increased cytotoxicity of oncolytic viruses was initially thought to be due to increased viral replication and, thus, increased tumour cell infection and oncolysis. However, data from a number of *in vitro* studies do not support this hypothesis, not only for GL-ONC1/GLV-1h68 but also for other OV [19–21, 26]. One area of recent research has been the role of GLV-1h68 in inducing apoptosis, with several studies demonstrating an increase in caspase 3 cleavage [6, 13, 19, 21]. This highlights the possibility that the primary mechanism of cell death may be apoptosis. However, the pathway of apoptosis induction and the effects of GLV-1h68 on the apoptotic signalling pathways have not been fully elucidated.

In these studies, we aim to determine the efficacy of combining oncolytic vaccinia virus with radiotherapy, investigate the mechanism of cell death of GLV-1h68 and to determine if this can be exploited for clinical use with external beam radiation therapy (EBRT) to improve clinical outcomes.

## RESULTS

### The viability of GLV-1h68 is not affected at clinically relevant doses of EBRT

**Figure 1:**
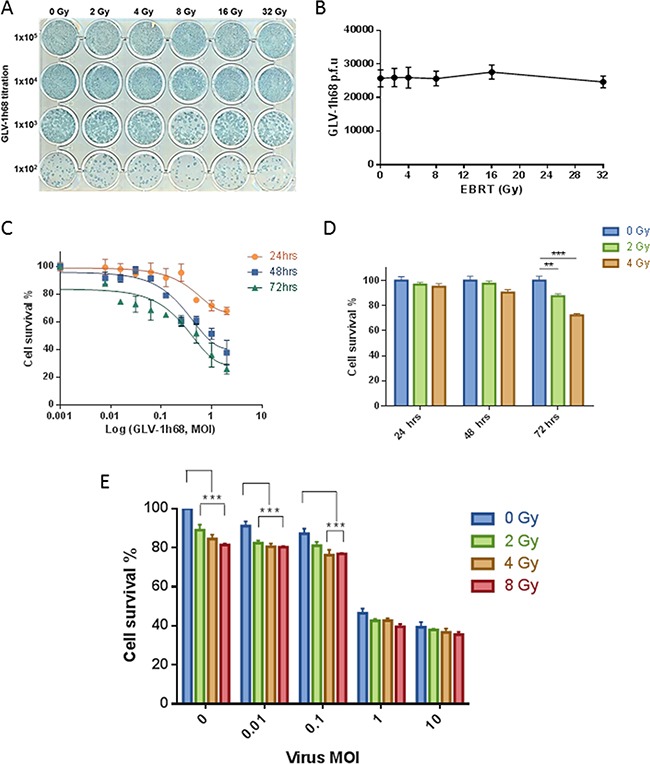
GLV-1h68 is not affected by clinically relevant doses of EBRT and shows enhanced cytoxicity against BN175 cells in combination with EBRT **A.** Photograph of viral plaque assay showing GLV-1h68 titration post-treatment with increasing doses of irradiation and **B.** line graph showing quantification of viral titres. MTT analysis of single agent and combination therapy of GLV-1h68 and EBRT against BN175 cells **C.** Line graph showing single agent efficacy of GLV-1h68, **D.** bar graph of single agent efficacy of EBRT and **E.** bar graph of cell survival at 72 hours with combination therapy (** = p value <0.01, *** = p value <0.001).

The direct effect of EBRT on the viability, replication competency and transgene expression of GLV-1h68 was assessed *in vitro*. GLV-1h68 was treated with increasing doses of radiation (0-32Gy). After exposure to radiation serial dilutions of virus were added to a confluent layer of CV1 cells in a 24-well plate. After 48 hours of incubation the viral plaques were detected using X-Gal staining (Figure 1A).

After triplicate repeats, the mean number of viral plaques per well for the non-irradiated virus was 2.57 X 10^4^ (95% CI 1.48 – 3.67 X 10^4^) compared to 2.47 X 10^4^ (95% CI 1.71 – 3.23 X 10^4^) after 32 Gy. After exposure to EBRT, no significant difference was found in the number of viral plaque forming units (p.f.u.), the macroscopic appearance of the plaques or the ability of GLV-1h68 to produce LacZ between the non-irradiated virus and any of the escalating radiation doses (One-way ANOVA; p= 0.9874) (Figure 1B).

### The cytotoxic effect of EBRT is enhanced when combined with GLV-1h68

The cytotoxicity of EBRT and GLV-1h68 alone, or in combination, was assessed using the BN175 cell line as a prelude to *in vivo* experiments. BN175 cells were treated with either GLV-1h68 at MOIs of 0.001 – 2 or EBRT between 0 to 4 Gy and incubated for 24, 48 or 72 hours.

Evaluation by MTT cell proliferation assay showed that GLV-1h68 had a cytotoxic effect on BN175 cells and that this increased in a time- and dose-dependent fashion (Figure 1C). At 24 and 48 hours, EBRT had no significant cytotoxic effect. However, at 72 hours, radiation alone showed significant efficacy at 2 Gy, mean survival 87 % (p < 0.01) and 4 Gy, mean survival 72 % (p < 0.001) compared to non-irradiated controls (Figure 1D).

In the combination therapy experiments, BN175 cells were treated with a range of viral doses and irradiated 6 hours post-viral treatment with 0, 2, 4 and 8 Gy. On analysis at 72 hours, the cytotoxicity of GLV-1h68 at MOI of 0.01 was significantly increased when combined with 2, 4 and 8 Gy compared to viral treatment alone and at an MOI of 0.1 the cytotoxicity was significantly increased with 4 and 8 Gy (Two-way ANOVA with Bonferroni post-test; p <0.001). The addition of radiation at higher viral MOIs (1 and 10) was not significant and did not further enhance the cytotoxicity effect seen in vitro (Figure 1E).

Comparative experiments using a viral MOI of 0.1 were conducted in a panel of human sarcoma cell lines. The combination of GLV-1h68 and radiation resulted in significantly increased cytotoxicity in one of three cell lines (Supplementary Figure S1).

### The combination of EBRT and GLV-1h68 is at least additive and at best synergistic on assessment by long-term clonogenic assay

**Figure 2:**
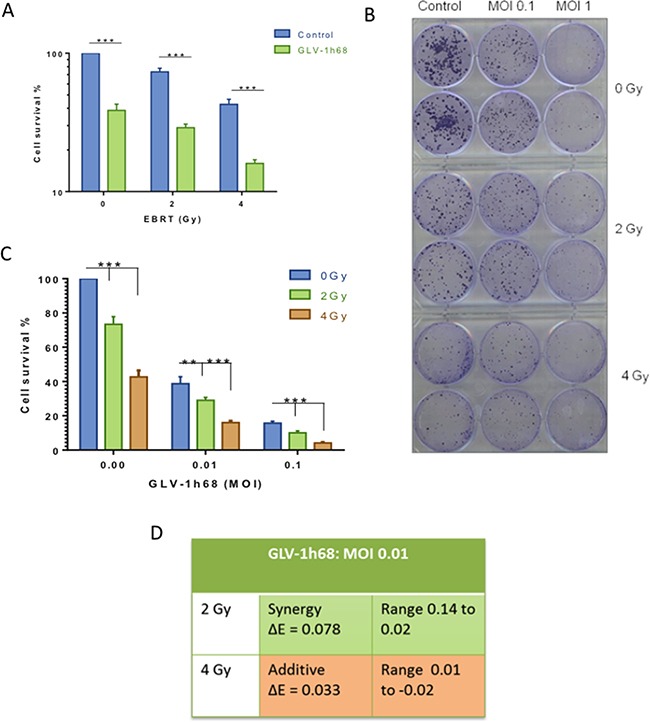
Assessment of combination therapy using colony formation assays revealed a synergistic cytotoxicity with GLV-1h68 and EBRT at 2 Gy **A.** Bar graph showing increased cytotoxicity with the addition of GLV-1h68 (MOI 0.01) to EBRT. **B.** photograph of clonogenic assay 6-well plates stained with crystal violet 10 days post-treatment. **C.** Bar Graph of the combination therapy with increasing viral MOI's and EBRT 2 and 4 Gy **D.** Bliss analysis of combination therapy (GLV-1h68 MOI 0.01, radiation at 2 and 4 Gy). (** = p value <0.01 and *** = p value <0.001).

Colony formation assays are the gold-standard method for assessing the effect of radiation *in vitro* [27]. Therefore, colony formation assays were used to test the efficacy of the different treatment regimens relative to the plating efficiency. The BN175 cell line showed sensitivity to radiation alone and in combination with GLV-1h68. Furthermore, this cytotoxicity increased in a dose-dependent fashion with statistical significance at both 2 and 4 Gy with a GLV-1h68 MOI of 0.1 and 0.01 (Two-way ANOVA with Bonferroni post-test; p <0.001), (Figure 2A, 2B and 2C).

The presence of synergy between therapies was tested using Bliss analysis. This demonstrated that combination therapy at 4 Gy had an additive effect and that the combination of 2 Gy with GLV-1h68 MOI 0.01 appeared synergistic (Figure 2D).

### The enhanced cytotoxic effect observed when combining EBRT and GLV-1h68 is not due to accelerated viral replication

To investigate if the observed synergy between EBRT and GLV-1h68 was due to increased viral replication, the titres of virus present after different treatment regimens was assessed by both qPCR, measuring A21L expression relative to the house-keeping gene 18S, and viral plaque assays (VPA).

**Figure 3:**
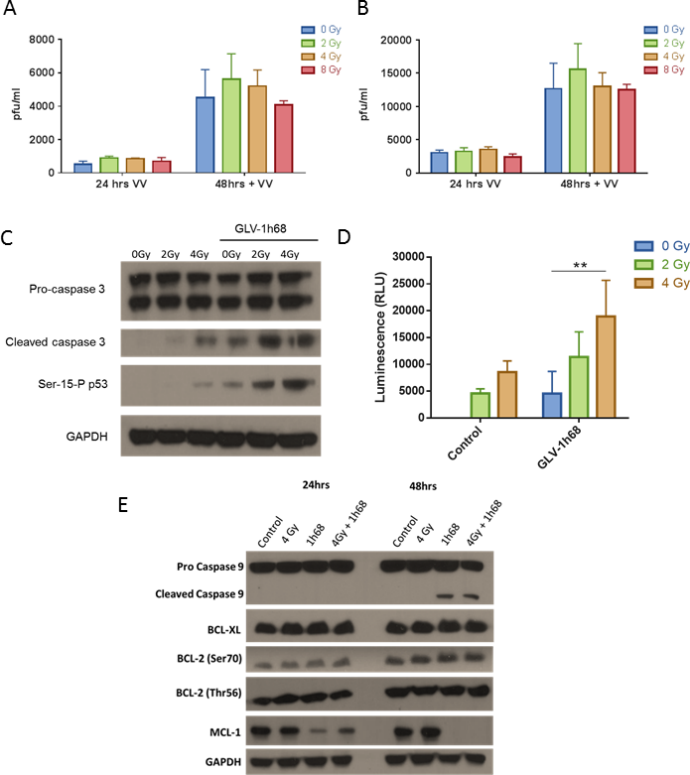
The enhanced cell kill observed in the BN175 cells after treatment with GLV-1h68 and EBRT is not due to increased viral replication but mediated via induction of intrinsic apoptosis and downregulation of the anti-apoptotic MCL-1 protein **A.** Viral plaque assay (VPA) investigating the recovery of live GLV-1h68 at progressive time points post-treatment with GLV-1h68 MOI 0.01 (VV) alone or in combination with radiation (0-8 Gy) and **B.** with GLV1h68 MOI 0.1. **C.** Western blot analysis of cleaved caspase 3 and Serine-15 phosphorylated p53 and **D.** Caspase-Glo assay of caspase 3/7 activity 48 hours post-combination therapy (** = p value <0.01). **E.** Western blot analysis of BN175 cells 48 hours after treatment with GLV-1h68 (MOI 0.1) and EBRT (4 Gy).

On assessment by VPA, the presence of live replication-competent GLV-1h68 was not significantly increased by radiation therapy at 2, 4 or 8 Gy, compared to non-irradiated controls. Conversely, viral replication was not abrogated by the effect of radiation alone, with a 5-fold increase in viral titres from 24 to 48 hours seen at all radiation doses (Figure 3A and 3B).

On qPCR, the quantity of GLV-1h68 genomes after the different treatment regimens was assessed at 6, 24 and 48 hours post-treatment. For all treatment groups, this showed an expected increase in genome copy numbers over the time course of the experiment. With the combination of GLV-1h68 and EBRT at 48 hours in comparison to virus alone, there was a statistically significant increase in viral copy numbers following 2 Gy irradiation (p <0.001). However, this significant increase was not found with the addition of 4 Gy (p >0.05) and at 8 Gy there was a statistically significant decrease in GLV-1h68 copy numbers compared to GLV-1h68 treatment alone (p <0.01) (Supplementary Figure S2). Taken together, these data suggest that the synergistic/additive effects of EBRT and GLV-1h68 are not due to altered viral replication.

### Combination therapy with GLV-1h68 and EBRT increases apoptotic cell death in a dose-dependent fashion

Having shown that the enhanced cytotoxicity between EBRT and GLV-1h68 was independent of viral replication, the mechanism of cell death was investigated. On western blot analysis, the addition of GLV-1h68 to EBRT, at 2 and 4 Gy, resulted in an increase in caspase 3 cleavage compared to EBRT therapy alone. Serine-15 is the primary target of the DNA damage response on the p53 protein [28] and was used as a marker of ionizing radiation-induced DNA damage [29, 30]. With EBRT alone, Serine-15 p53 phosphorylation was only apparent at 4 Gy but did correlate with the presence of cleaved caspase 3. Interestingly, GLV-1h68 alone was able to induce Serine 15 phosphorylation of p53 and this significantly increased when combined with EBRT at 2 and 4 Gy compared to EBRT alone. This coincided with an increase in the level of cleaved caspase 3 (Figure 3C).

To validate and quantify the apoptotic response to combined therapy, a Caspase-Glo assay was used to measure the levels of caspase 3/7 cleavage after different treatment regimens. Treatment with radiation increased cleaved caspase 3/7 induction at 2 and 4 Gy. Both GLV-1h68 and EBRT alone induced caspase 3/7 cleavage and this induction significantly increased with the combination of GLV-1h68 and 4 Gy compared to either agent alone (Two-way ANOVA with Bonferroni post-test; p <0.01), (Figure 3D).

### Combination therapy activates the intrinsic apoptotic pathway and downregulates anti-apoptotic proteins

Apoptosis as the mechanism of cell death was further evaluated in the BN175 cell line using western blot analysis. Treatment with GLV-1h68 alone induced cleavage of caspase 9 at 48 hours post-treatment, which was not seen with EBRT alone and no further increase in cleaved caspase 9 was seen with the combination of GLV-1h68 and EBRT. The identification of cleaved caspase 3 and 9 at 48 hours after treatment suggests that GLV-1h68 may cause cell death by intrinsic pathway-mediated apoptosis (Figure 3E).

To elucidate further the mechanism of apoptosis, the BCL-2 family of proteins was studied to ascertain if GLV-1h68 infection had an effect on the critical negative regulators of apoptosis. On western blot analysis, untreated BN175 cells appeared to have high baseline levels of all the inhibitors of apoptosis tested (BCL-XL, Total BCL-2, BCL-2 Ser70, BCL-2 Thr56 and MCL-1). The levels of all of these proteins, except for MCL-1, were unchanged at 24 and 48 hours post-treatment with either, GLV-1h68, EBRT or combination therapy.

MCL-1, an early critical negative regulator of apoptosis, which ultimately prevents the activation of mitochondrial cytochrome C release [31, 32] was present at high baseline levels in BN175 cells. At 24 hours post-treatment with GLV-1h68, the levels decreased compared to untreated or EBRT only treatment and at 48 hours MCL-1 was undetectable following GLV-1h68 treatment alone and combination therapy (Figure 3E).

**Figure 4:**
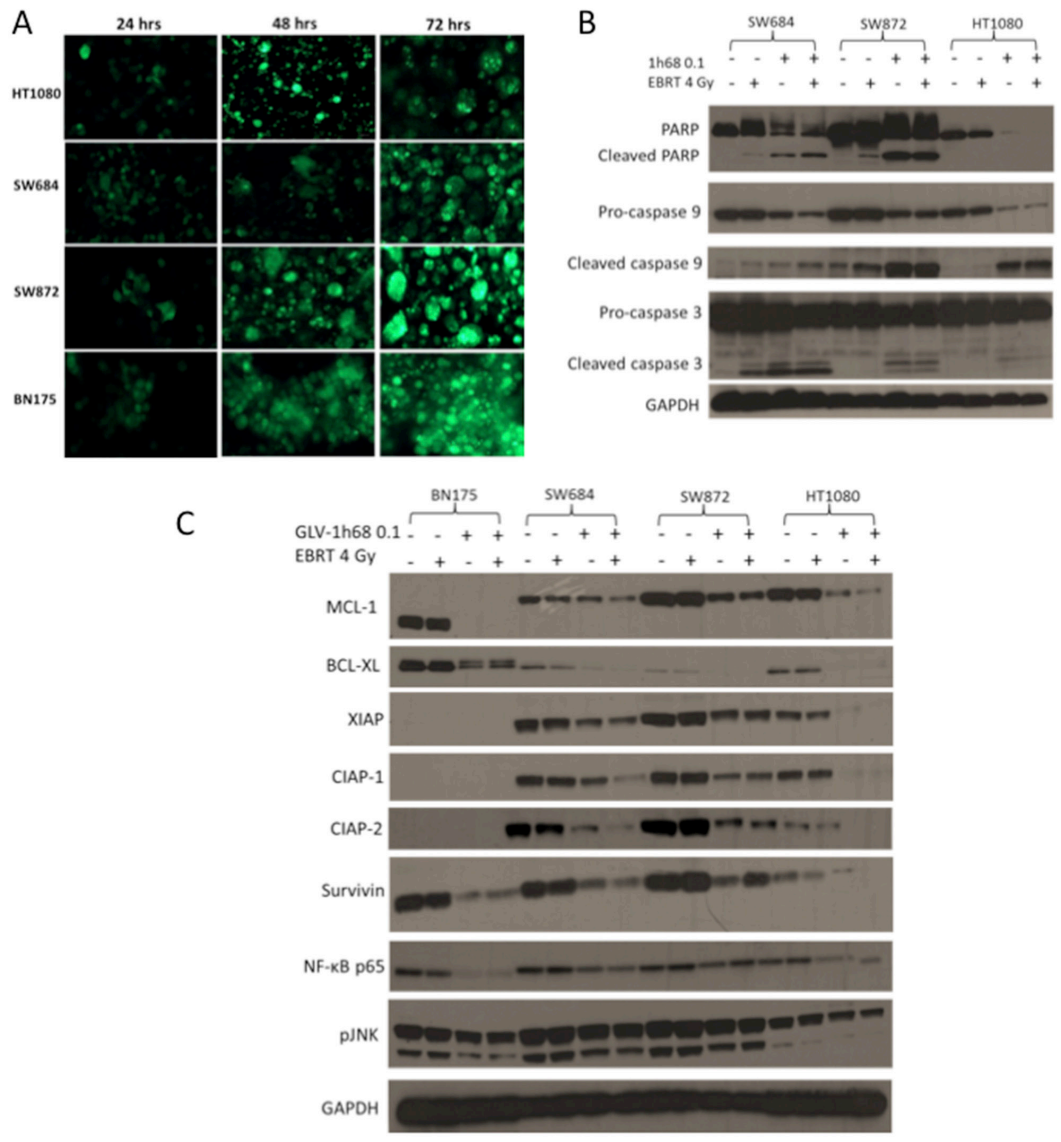
Efficient infection of cells, with the GFP producing GLV-1h68, leads to apoptotic cell death due to the loss of the anti-apoptotic MCL-1 protein and the inhibitors of apoptosis (IAPs) **A.** GFP transgene expression in the rat BN175 cell line and a panel of human sarcoma cell lines (HT1080, SW684 and SW872) at 24, 48 and 72 hours after treatment with GLV-1h68 (MOI 1). **B.** Western blot analysis of 3 human sarcoma cell lines after treatment with GLV-1h68 (MOI 0.1) and EBRT (4 Gy). **C.** Western blot analysis of the expression of anti-apoptotic BCL2 proteins, IAPs and downstream pro-survival signalling pathways 48 hours after treatment with GLV-1h68 (MOI 0.1) and EBRT (4 Gy) alone or as combination therapy. The MCL-1 protein was consistently detected at 40kDa in the human cell lines and at 37kDa in the BN175 rat cell line.

A panel of human sarcoma cell lines were used to validate the observed decrease of MCL-1 in the rat fibrosarcoma cell line and to determine if the *in vitro* findings translated to human sarcomas and potentially to patients. The SW872 liposarcoma, SW684 fibrosarcoma and HT1080 fibrosarcoma cell lines were kindly provided by Prof J. Shipley. The cytotoxic effect of GLV-1h68 single agent therapy on these cell lines has previously been reported [13]. The ability of GLV-1h68 to infect and produce efficient transgene expression in all the cell lines was tested. Cells were plated in a confluent layer in individual wells on a 24-well plate and assessed with fluorescence microscopy at 24, 48 and 72 hours for the expression of viral GFP. GFP transgene expression was consistently detectable at 24 hours post-infection with GLV-1h68 (MOI 1) in all cell lines and intensified at 48 and 72 hours (Figure 4A).

In all three human sarcoma cell lines (SW684, SW872 and HT1080), the induction of caspase 3, 9 and PARP was investigated. Cleavage of PARP (or loss of full length PARP), caspase 9 and caspase 3 was seen after treatment with GLV-1h68 alone and in combination with EBRT in all cell three lines. This was notably enhanced compared to controls or EBRT alone (Figure 4B).

In the human sarcoma cell lines, the anti-apoptotic proteins MCL-1 and BCL-XL were not affected by EBRT alone. However, after treatment with virus alone or in combination with EBRT, the levels of the anti-apoptotic protein BCL-XL were considerably reduced. The level of MCL-1 decreased substantially with GLV-1h68 therapy alone in the human sarcoma cell lines and showed a further decrease when combined with EBRT (Figure 4C).

The inhibitors of apoptosis (IAP) family of proteins were also examined. The primary antibodies used (IAP Family Antibody Sampler Kit #9770, Cell Signalling) were all human-specific except for Survivin. Therefore, only levels of Survivin were detectable in the rat BN175 cell line. All of the IAP proteins tested were expressed in the untreated human sarcoma cell lines.

In the SW684 cell line, the protein levels of all of the IAPs tested (XIAP, CIAP-1, CIAP-2 and Survivn) were unaffected by EBRT alone but decreased with GLV-1h68 single agent therapy, with a further decrease after combination therapy. In the SW872 cells, EBRT alone again had no effect on the level of any of the IAP proteins. GLV-1h68 decreased the expression level of all IAP proteins but with no further reduction when combined with EBRT. GLV-1h68 appeared to have the greatest effect on the N-ras mutant HT1080 cells, in which the levels of XIAP, CIAP-1, CIAP-2 and Survivin decreased with GLV-1h68 alone and were completely undetectable after combination therapy with GLV-1h68 and EBRT.

XIAP is reported to activate JNK and NF-κB pro-survival signalling within the cell [33]. Therefore, to elucidate if the down-regulation of XIAP resulted in downstream effects, the levels of NF-κB and pJNK were tested. In all of the four cell lines, there was a decrease in the levels of both NF-κB p65 and pJNK with GLV-1h68 treatment alone or in combination with EBRT Figure 4C.

In order to determine whether down-regulation of expression was limited to anti-apoptotic proteins following treatment with GLV-1h68 and EBRT, the levels of the pro-apoptotic proteins Bax and Bak were tested. Bak was not expressed in the BN175 cell line and its expression in the human sarcoma cell lines was not affected by treatment with GLV-1h68 and EBRT alone or in combination. The expression of Bax was not found to alter in the BN175, SW872 or HT1080 cell lines and appeared to be upregulated following treatment with GLV-1h68, alone or in combination with EBRT, in the SW684 cell line (Supplementary Figure S3).

### Combination therapy with GLV-1h68 delivered by ILP and EBRT resulted in prolonged survival compared to controls or single agent therapy

The *in vivo* effects of the combination of EBRT and GLV-1h68 delivered by ILP were studied in an orthotopic model of advanced extremity sarcoma. Animals were engrafted with 1 x 10^7^ BN175 cells prior to undergoing either ILP with GLV-1h68 alone or femoral artery and vein ligation, followed by EBRT at 48 and 72 hours post-procedure.

**Figure 5:**
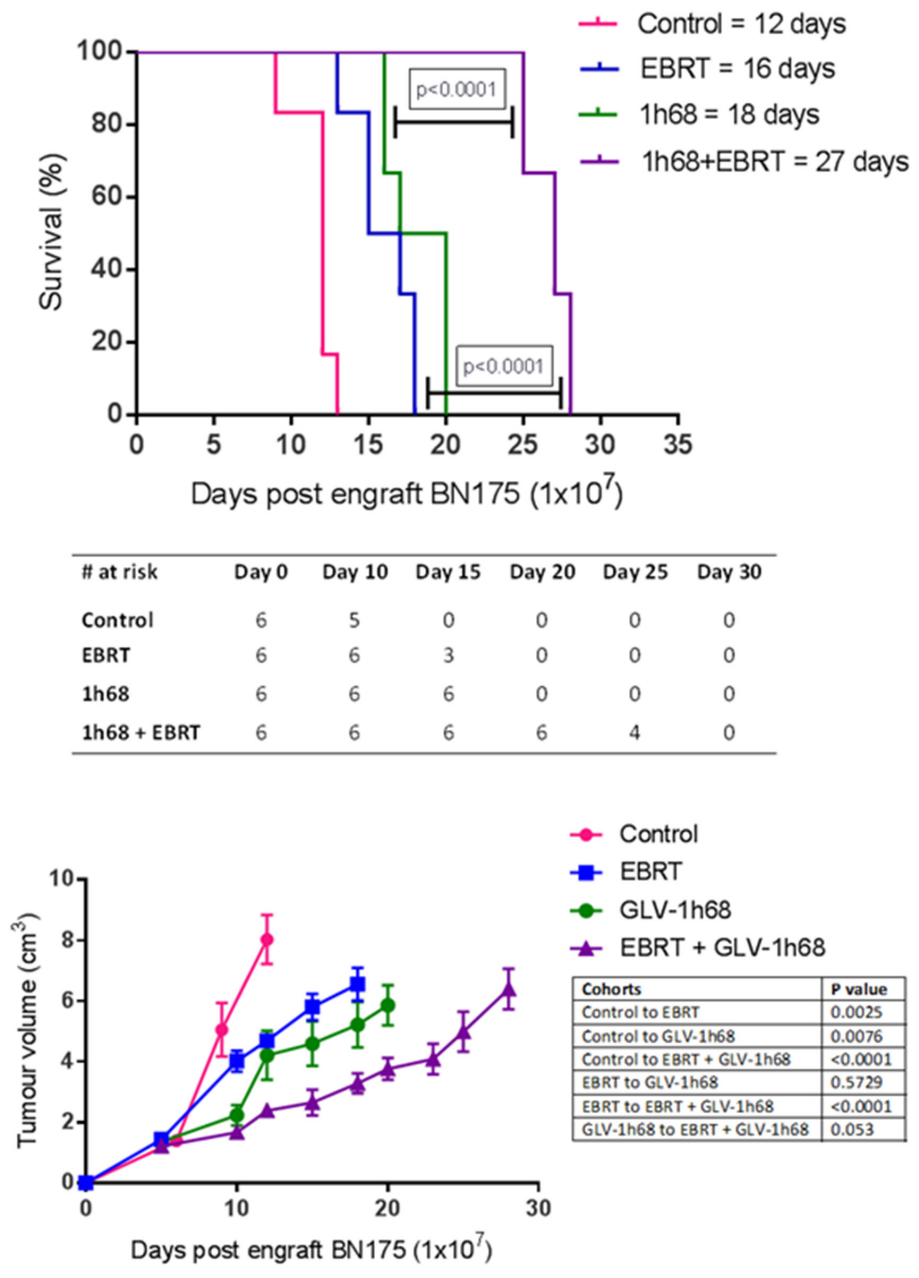
In an aggressive sarcoma model combination therapy with GLV-1h68 delivered by ILP and adjuvant EBRT (13 Gy/2 fractions) prolongs survival and delays tumour growth in Brown Norway rats, bearing BN175 tumours on the left hind limb **A.** Kaplan Meier curve of survival in each treatment cohort with median time to humane endpoint displayed in group legend. **B.** Table displaying surviving number of animals in each cohort at progressive time points and **C.** tumour growth volumes as determined by external calliper measurement.

A significant delay in tumour growth was seen in animals treated with either EBRT or GLV-1h68 single agent therapy, with a median increase in time to humane endpoint, compared to controls, of 4 and 6 days (33% and 50%), (one-way ANOVA, Tukey's post-hoc test p <0.01 and p <0.001), respectively. Furthermore, the combination of GLV-1h68 and EBRT was found to significantly increase time to humane endpoint compared to either agent alone (one-way ANOVA, Tukey's post-hoc test p <0.0001). Median times to humane endpoints were 12, 16, 18, 27 days in control, EBRT alone, GLV-1h68 alone and GLV-1h68 and EBRT groups, respectively (Figure 5A, 5B and 5C).

Single agent and combination therapy was well tolerated with no evidence of cutaneous or systemic toxicity in the any of the animals treated.

## DISCUSSION

Previous work with an orthotopic model of extremity sarcoma has demonstrated the potential of OV when delivered by ILP. The addition of OV to a standard ILP protocol resulted in tumour growth delay and prolonged survival [13]. However, local disease control was not achieved and modifications of this treatment strategy to maximise efficacy were sought. Radiotherapy plays a vital role in the management of extremity sarcoma and has been shown to be synergistic with OV in other tumour models [6, 18–21, 23]. The purpose of this study was to determine if the combination of OV with radiotherapy is an effective treatment strategy when combined with ILP and to investigate the mechanistic interactions that may be exploitable for clinical benefit.

As GLV-1h68 is a double stranded DNA virus [34], and so potentially vulnerable to the damaging effects of ionising radiation, we first determined the effects of EBRT on viral viability. GLV-1h68 was found to be resistant to radiation at doses far higher than would be used in the clinic, up to a maximum of 32 Gy in a single fraction. As such, in both the pre-clinical and clinical setting, EBRT may be scheduled either before or after delivery of vaccinia virus with no effect on therapeutic efficacy. This is of particular significance for downstream clinical translation, not least of all because the natural sequence for combining these modalities would be intravascular viral delivery (during ILP) followed by EBRT.

Not only is GLV-1h68 resistant to EBRT, but also the combination of these modalities was found to be at least additive and possibly synergistic. A small but significant increase in cytotoxicity was seen with the combination of GLV-1h68 at both 2 and 4 Gy on MTT assays. Furthermore, a synergistic effect with the addition of GLV-1h68 at 2 Gy and an additive effect at 4 Gy were noted on clonogenic assays, the gold-standard for assessing the effects of ionising radiation *in vitro* [27].

Additive or synergistic effects following the combination of OV with radiation have been demonstrated in a number of pre-clinical models and the mechanisms responsible are likely to be multifactorial. One putative mechanism is enhanced oncolysis secondary to a radiation-induced increase in viral replication [25]. However, there are several conflicting studies in the literature concerning OV cytotoxicity and correlation to viral replication. A number of studies with a range of different viruses have suggested that enhanced lytic cytotoxicity is due to increased viral replication following combination treatment with either radiation or cytotoxic chemotherapy [35–37]. However, others have reported no change or even a reduction in viral replication in combination regimens with radiation or chemotherapy [6, 21, 38–42]. Our experiments have also shown enhanced cytotoxicity in the absence of increased viral replication, adding to the existing evidence of an additive interaction between OV and radiation that is independent of viral replication.

**Figure 6:**
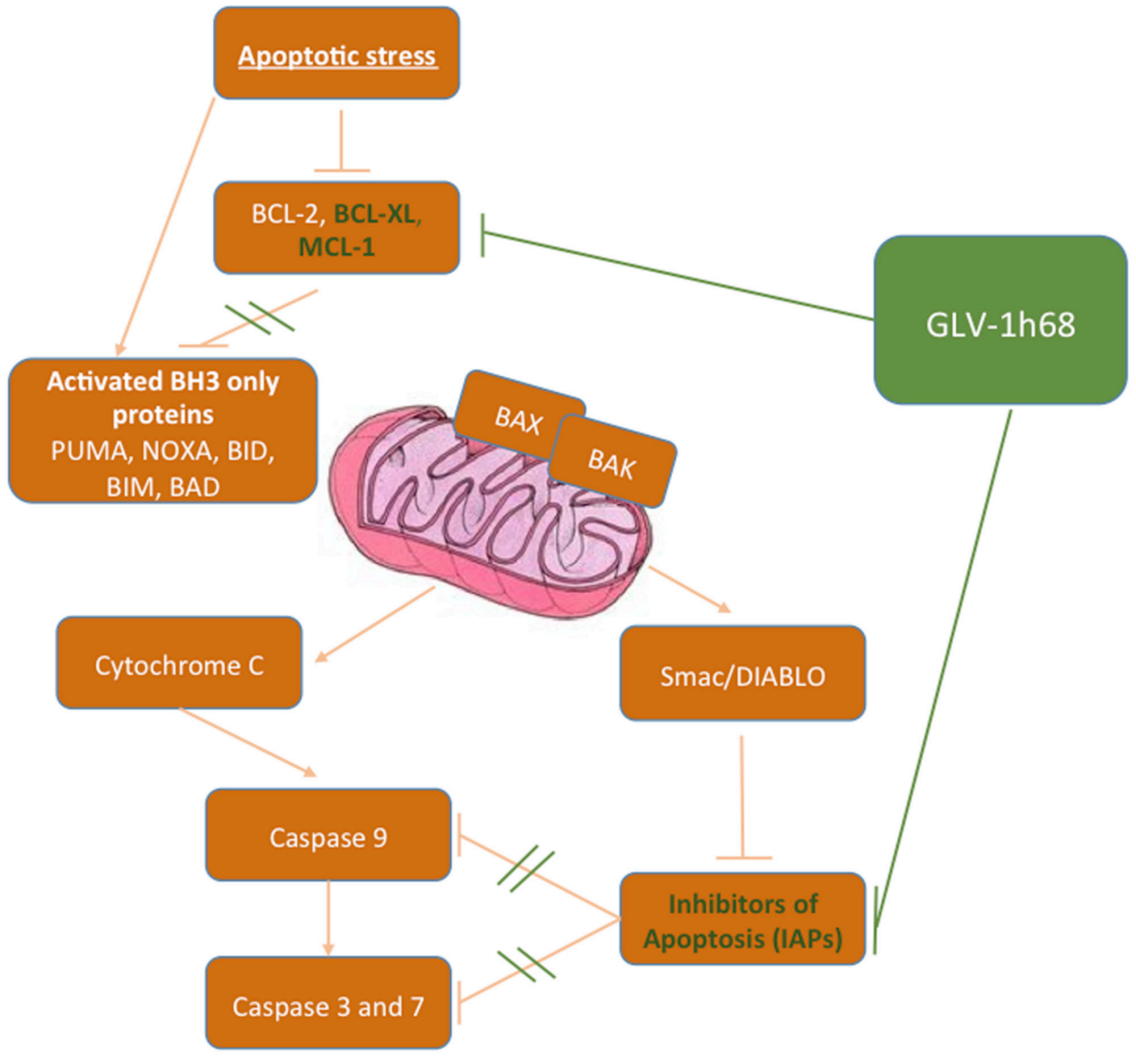
Simplified schematic demonstrating the postulated interactions of GLV-1h68 on the intrinsic apoptotic pathway proteins When activated by an apoptotic stimuli the intrinsic apoptosis pathway causes accelerated degradation of the anti-apoptotic BCL-2 proteins (BCL-2, BCL-XL, BCL-W and MCL-1) resulting in activation of the pro-apoptotic BH3 only proteins (PUMA, NOXA, BID, BIM, BAD, BAX and BAK). This results in initiation and propagation of the effector caspases 3 and 7. In cancer cells apoptosis is circumvented through overexpression of the anti-apoptotic BCL-2 proteins and the IAPs. Treatment with GLV-1h68 restores the cells ability to undergo apoptosis by inhibiting the overexpression of these negative regulators of apoptosis.

The precise mechanisms of cell death following infection with GLV-1h68 remain to be fully elucidated but recent studies have suggested an induction of apoptosis may be a critical component [6, 17, 19]. Our data support these findings, demonstrating a significant increase in caspase cleavage in sarcoma cell lines exposed to this virus. Infection with GLV-1h68 was also shown to downregulate a number of anti-apoptotic proteins, such as MCL-1, BCL-XL and the IAP family. Although vaccinia virus has also been shown to result in a reduction in a range of cellular transcripts [43], a reduction in the pro-apoptotic proteins Bak and Bax was not seen. In fact, in one cell line Bax was up-regluated after treatment with GLV-1h68. These data suggest that infection with GLV-1h68 preferentially downregulates anti-apoptotic proteins, resulting in an overall shift in protein expression within the cell tipping the balance in favour of apoptosis (Figure 6). A further decrease in the expression of anti-apoptotic proteins was noted when GLV-1h68 was combined with EBRT compared with either modality alone. This may represent a radiosensitising effect following viral infection. Ionising radiation results in DNA damage, which, if irreparable, triggers cell death via apoptosis. Viral proteins have the potential to interact with a number of intracellular signalling pathways regulating apoptosis that are triggered by radiation, with vaccinia implicated in manipulation of the TNF/JNK, MAPK and Akt pathways [6, 44–46]. It may be that interference with one or a number of these pathways are responsible for the additive effects seen, and the precise nature of this interaction warrants further study.

Despite engendering a pro-apoptotic state in our experiments, vaccinia virus is also known to encode several proteins that prevent apoptosis [47]. Furthermore, in other preclinical models, infection with vaccinia virus has been shown to be capable of inducing necrosis and necroptosis [48–51]. The effects of combining OV and radiation on the relative proportion of cells dying from necrosis or necroptosis in our cell lines is unknown and further work will be directed at investigating whether the promotion of apoptosis is accompanied by a reduction in cell death by other means.

Our *in vitro* findings translated into a therapeutic effect *in vivo*. Using this aggressive limb sarcoma model, the combination of delivering GLV-1h68 by ILP and EBRT significantly improved survival and proved to be far superior to single agent therapy. This effect was beyond what would be expected from a simply additive effect. This is promising as it means that OV and radiotherapy can potentially be combined with currently used ILP therapeutics (melphalan and TNF-α) and/or surgical resection in an attempt to produce a curative therapeutic regimen in preclinical models. Importantly, this would have significant translational potential in the clinic.

## MATERIALS AND METHODS

### *In vitro* studies

#### Cell lines

The BN175 rat sarcoma cell line was donated by Prof. A. Eggermont. This cell line is tumorigenic in Brown Norway rats [52]. Cells were passaged in Dulbecco's Modified Eagle's Medium (DMEM), supplemented with 5% heat-inactivated foetal calf serum (FCS), 2.5% L-glutamine and 1% penicillin/streptomycin. The CV-1 monkey kidney cell line was obtained from existing laboratory stocks and passaged in standard Dulbecco's Modified Eagle's Medium (DMEM), with 10% FCS, 2.5% L-glutamine and 1% penicillin/streptomycin. The HT1080, SW684 and SW872 human sarcoma cell lines were obtained by donation from Dr Janet Shipley and cultured in similarly modified DMEM media. Cells were cultured at 37 °C in an incubator maintaining a 10% carbon dioxide atmosphere.

#### Oncolytic vaccinia virus

GLV-1h68 was produced and provided by Genelux Corporation (San Diego, USA) as described previously [12]. Briefly, three expression cassettes encoding *Renilla* luciferase-GFP fusion protein, β-galactosidase, and β-glucuronidase were recombined into the *F14.5L*, *J2R* and *A56R* loci, respectively, of the parental Lister strain, LIVP, virus genome.

#### External beam radiotherapy (EBRT)

All irradiations were performed using an Orthovoltage X-ray source (320/250 kV; serial no: 20090606) (AGO X-Ray Ltd, Reading, UK) at 250 kVp and at a dose rate of approximately 0.6 Gy/min, as measured directly by a UNIDOS^©^E Universal Dosimeter (PTW, Grantham, UK). Cells were irradiated in 6-, 24- or 96-well plates (Nunc) in single fractions up to 8 Gy. Eppendorf tubes containing GLV-1h68 at 1 x 10^5^ p.f.u were irradiated at 0, 2, 4, 8, 16 and 32 Gy.

#### 3-(4,5-dimethylthiazol-2-yl)-2,5-diphenyltetrazolium bromide (MTT) assay

Estimation of cell proliferation after exposure to cytotoxic agents alone or in combination was performed using the 3-(4,5-dimethylthiazol-2-yl)-2,5-diphenyltetrazolium bromide (MTT) assay [53]. Cells were plated in a clear-walled 96-well plate (Nunc, Denmark) at a density of 2500 cells per well. GLV-1h68 was added after 16 hours and the cells were irradiated 6 hours after viral infection. MTT reagent was added at the time points indicated. The absorbance of a solution (in dimethyl sulfoxide, DMSO) of the reduced MTT was read on a plate reader (Victor 2, Perkin Elmer, Mass., USA) at a wavelength of 550 nm. Results were normalised to a control population of untreated cells. Cell kill was calculated as (100 – surviving fraction) %.

#### Viral plaque assay (VPA)

BN175 cells were plated at 1 × 10^5^ per well in 24-well plates. Plates were treated with GLV-1h68 (MOI 0.01 or 0.1) and then incubated for 6 hours before being irradiated at 0, 2, 4 or 8 Gy. Supernatant and cells were collected at 24 and 48 hours post-infection and the viral particles from the infected cells were released by 3 freeze-thaw cycles and then stored at -80 °C.

CV-1 cells were grown to confluence on 24-well plates. The supernatants were thawed, and then incubated on the CV-1 cells. After 48 hours of incubation, virus detection was carried out on the cell monolayer. Briefly, cells were fixed with 2% formaldehyde/0.2% glutaraldehyde solution for 5 minutes, then washed in PBS and stained for 4 hours with X-Gal staining buffer and X-Gal (CalBioChem, Merck KGaA, Germany) (1:100). Finally, they were washed with UF water and dried.

Macroscopic X-gal-stained viral plaques were counted manually. Detected levels were normalised according to the proportion of surviving cells, as determined by sulphorhodamine B (SRB) assay carried out simultaneously.

In a second experiment to determine the potential cytotoxic effect of EBRT on GLV-1h68, 1 x 10^5^ plaque-forming units (PFU) of virus in 1 mL of PBS, was irradiated in Eppendorf tubes at 0-32 Gy before being added to confluent CV-1 cells. Virus detection was carried out after 48 hours of incubation and the number of viral plaques counted manually.

#### Clonogenic assay

BN175 cells were plated at 5 × 10^5^ cells in a T25 flask and then incubated at 37°C. After 16 hours, the cells were treated with GLV-1h68 MOI 0.01 or 0.1 and left in the incubator for a further 48 hours. The cells were then washed, trypsinized and counted using a haemocytometer and added to 6-well plates, at seeding densities from 200 to 800 cells per well, in 5 mL of medium with 10% FCS added to each well. Sixteen hours after plating, cells were irradiated at 0, 2, 4, 6 or 8 Gy, and then incubated at 37°C.

After 10 days, cells were fixed, stained and the number of colonies per well counted. The plating efficiency of the experiment was determined from the control wells and calculated for every experiment. The surviving fraction of cells after treatment was calculated by normalising the number of colonies per well to the plating efficiency.

#### Bliss analysis

To investigate the cytotoxic effect Bliss independence analysis was used as previously described [54–57]. Briefly, this methodology uses the formulae E_IND_ = E_A_ + E_B_ − E_A_ × E_B_ and ΔE = E_OBS_ − E_IND_ where: E_A_ and E_B_ are the fractional effect of factors A and B, respectively; E_IND_ is the expected effect of an independent combination of factors; E_OBS_ is the observed effect of the combination. If ΔE and its 95% confidence interval (CI) are >0, synergy has been observed. If ΔE and its 95% CI are <0, antagonism has been observed. If ΔE and its 95% CI contain 0 then the combination is independent. A recently published modification of Bliss independence analysis could not be used because of the number of variables within our dataset [58].

#### qPCR

Samples were collected as per viral plaque assay methods. A21L expression qPCR using the Genelux GL-LC1 VV-A21L kit was used for quantitative detection of the vaccinia A21L gene. DNA was prepared from the lysate of BN175 cells following infection with GLV-1h68 (MOI 0.1) and irradiation (0, 2, 4, 6 or 8 Gy) at 6 hours post-infection and collected at 6, 24 and 48 hours post-treatment. A21L specific primers were used (forward: 5′-CGTAAACTACAAACGTCTAAACAAGAA-3′ and reverse: 5′-CCT GGTATATCGTCTCTATCTTTATCAC-3′). The 18S rRNA of human/rat genomic DNA (18S) was used as a reference to determine the copy number ratio of virus.

#### Western blot analysis

Cells were plated at 5 × 10^5^ in 60 mm dishes. Following various treatments, cells were harvested at 48 hrs in ice-cold PBS, pelleted and resuspended in radioimmunoprecipitation assay buffer (50 mM Tris (pH 7.5), 150 mM NaCl, 1% NP40, 0.5% sodium deoxycholate, and 0.1% SDS) with protease inhibitors (Roche Diagnostics GmbH, Mannheim, Germany), 1 mM sodium orthovanadate (Sigma), and 10 mM sodium fluoride. Cells were then lysed by snap-freezing on dry ice and then allowing the lysate to thaw on ice for 10 minutes. The lysate was then centrifuged at 13,200 rpm/4°C for 20 minutes to remove cell debris. Protein concentration was determined using the BCA protein assay (Pierce, Rockford, IL). Thirty μg of each protein sample were resolved on SDS-polyacrylamide gels (10-12%) and transferred to a polyvinylidene difluoride Hybond-P membrane. Immunodetections were performed using procaspase, cleaved caspase 3, cleaved caspase 9, phospho-p53 (Ser15), P-JNK (Thr^183^/Tyr^185^), Pro-survival Bcl-2 family antibody sampler kit, IAP family antibody sampler kit (Cell Signalling Technology), PARP and NF-KB (p65) (Santa Cruz Biotechnology Inc) rabbit polyclonal antibody in conjunction with a horseradish peroxidase (HRP)-conjugated anti-rabbit secondary antibody (GE-Healthcare). Equal loading was assessed using glyceraldehyde-3-phosphate dehydrogenase-GAPDH (Cell Signaling). The Super Signal chemiluminescent system (Pierce) or Immobilon Western chemiluminescent HRP substrate (Millipore) were used for detection.

#### Caspase-Glo assay

The Caspase-Glo 3/7 Assay (Promega) was used according to the manufacturer's instructions to determine the relative levels of Caspase 3/7 activation 48 hours post-infection with GLV-1h68 (MOI 0.1) +/- radiation (2 or 4 Gy). Detected levels were normalised according to proportion of surviving cells, as determined by a duplicate MTT assay carried out simultaneously.

### *In vivo* studies

#### Animals

Inbred, specific pathogen-free adult male Brown Norway rats weighing between 225 and 275 g were obtained from Charles River (Margate, UK). They were housed in compliance with all relevant regulatory requirements and fed standard chow and water *ad libitum*.

#### Tumour response experiments

All *in vivo* experiments were performed with bioethical approval from the British Home Office. Animals were randomly allocated to one of four treatment groups, containing six rats per group. Control, EBRT 13 Gy in two fractions, Isolated limb perfusion (ILP) with GLV-1h68 (1 x 10^7^ PFU) and ILP with GLV-1h68 (1x 10^7^ PFU) and adjuvant irradiation at 13 Gy in two fractions.

BN175 cells (1 x 10^7^ cells in 500 μL PBS) were injected into the left hind limb of the rat. ILP was performed on day 6 after cell injection. The ILP technique was a modified version of a previously reported method [59]. Animals underwent either femoral artery and vein ligation (controls and EBRT only) or ILP with GLV-1h68 1 x 10^7^ plaque forming units (pfu). Therapeutic perfusion was performed for 13 minutes and washout for 2 minutes.

Rats were irradiated, on both day 8 and 9 post-tumour engraftment to a total dose of 13 Gy. Before therapeutic tumour irradiation, rats received an intraperitoneal injection of a 1:1:2 solution of hypnorm (Janssen Pharmaceutical Ltd.), hypnovel (Roche), and sterile water. The resulting mixture contains 1.25 mg/mL midazolam, 2.5 mg/mL fluanisone and 0.079 mg/mL fentanyl citrate and is administered as a single intraperitoneal injection of 1.5 mL/kg bodyweight and titrated as required to a maximal dose of 3 mL/kg bodyweight. Anaesthetised animals were placed in a custom-made 6 mm lead shield. The shield holds the leg abducted from the midline, such that the tumour may be irradiated through a window, whilst minimising irradiation of surgical wound site, visceral or other structures. To quantify the radiation dose delivered to the tissues a universal dosimeter was placed adjacent to the tumour during irradiation.

#### Tumour growth and humane endpoints

Tumour growth was assessed every 48 hours by direct calliper measurement in two orthogonal; dimensions. Tumour volume was calculated using the formula: Volume = ½(width^2^ x breadth). Animals were culled at a pre-specified humane endpoint of 2 cm maximum tumour diameter. An additional humane endpoint of >10% weight loss was also defined but not reached in this study.

### Statistical analysis

Simple and descriptive statistical analysis was performed using Microsoft Excel (Microsoft Inc, Washington, USA). Kaplan-Meier survival analysis and group analysis, including ANOVA, was performed using GraphPad Prism software (GraphPad Software Inc, California, USA). Statistical significance was defined as a p-value of less than 0.05. Error bars on all graphs represent standard error of the mean.

## SUPPLEMENTARY MATERIALS FIGURES


