# Supplementary material for: Oncolytic vaccinia virus combined with radiotherapy induces apoptotic cell death in sarcoma cells by down-regulating the inhibitors of apoptosis
Source: Oncotarget. 2016 Oct 22;7(49):81208–22. doi: 10.18632/oncotarget.12820 (PMC5348387; doi:10.18632/oncotarget.12820)
Supplement: Supplementary file 1 [file oncotarget-07-81208-s001.pdf]

Oncolytic vaccinia virus combined with radiotherapy induces apoptotic cell death in sarcoma cells by down-regulating the inhibitors of apoptosis

SUPPLEMENTARY FIGURES

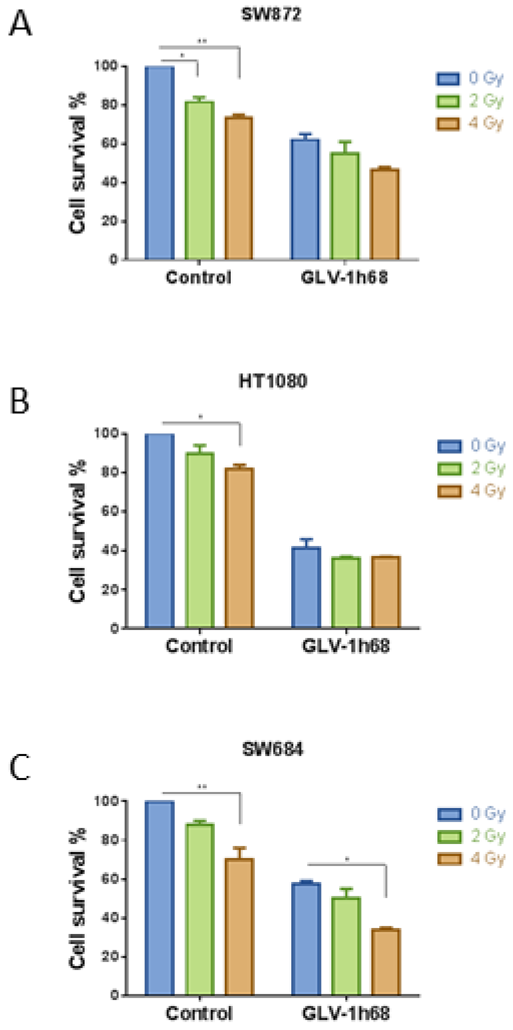

Supplementary Figure S1: Enhanced cytotoxicity with double combination therapy (GLV-1h68 and EBRT) in a panel of human sarcoma cell lines. MTT results for combination therapy after 72 hours in SW872 **A.**, HT1080 **B.** and SW684 **C.** sarcoma cells. GLV-1h68 group were treated with MOI 0.1.

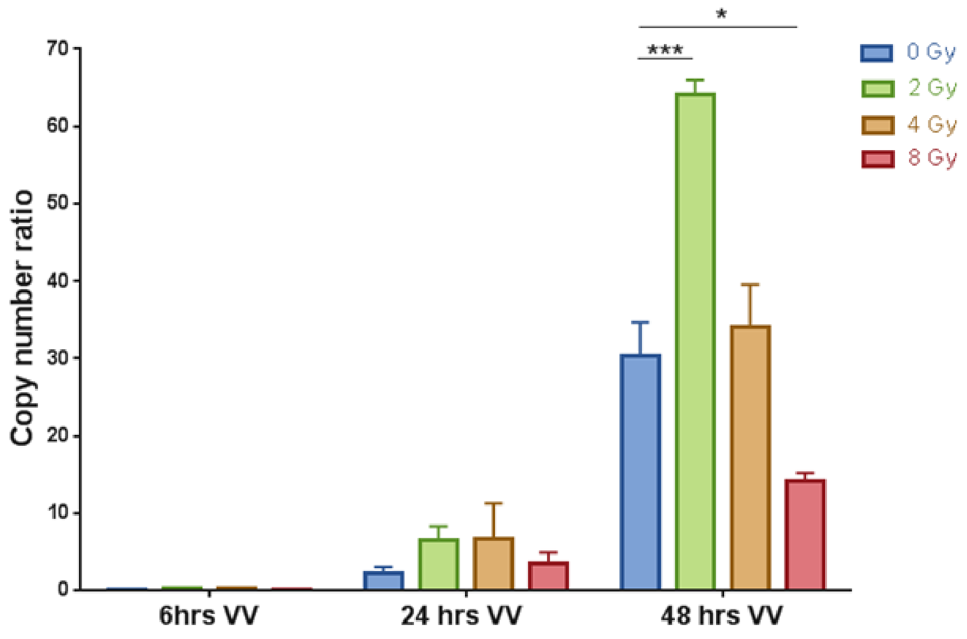

Supplementary Figure S2: The enhanced cell kill observed in the BN175 cells after treatment with GLV-1h68 and EBRT is not due to increased viral replication. Analysis by qPCR, the number of GLV-1h68 genomes present were quantified, using the vaccinia A21 gene, after different treatment regimens and progressive time points then normalised to the total DNA present using house-keeping gene 18S.

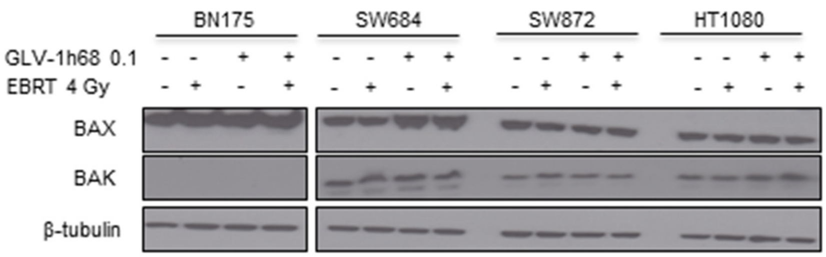

Supplementary Figure S3: Treatment with GLV-1h68 and EBRT does not downregulate pro-apoptotic proteins. Western blot analysis of the expression of the pro-apoptotic proteins Bax and Bak, 48 hours after treatment with GLV-1h68 (MOI 0.1) and EBRT (4 Gy) alone or as combination therapy. No difference was noted in the expression of Bak with an increase in the expression of Bax with GLV-1h68 or combination therapy in the SW684 cell line.
